# ASFT-Transformer: A Fast and Accurate Framework for EEG-Based Pilot Fatigue Recognition

**DOI:** 10.3390/s25196256

**Published:** 2025-10-09

**Authors:** Jiming Liu, Yi Zhou, Qileng He, Zhenxing Gao

**Affiliations:** 1 College of Civil Aviation, Nanjing University of Aeronautics and Astronautics, Nanjing 211106, China; ljm@nuaa.edu.cn (J.L.); sx2220002@nuaa.edu.cn (Q.H.); 2 College of General Aviation and Flight, Nanjing University of Aeronautics and Astronautics, Nanjing 211106, China; yzhou96@nuaa.edu.cn

**Keywords:** electroencephalography, fatigue recognition, feature selection, transformer-based model, flight simulator training

## Abstract

Objective evaluation of pilot fatigue is crucial for enhancing aviation safety. Although electroencephalography (EEG) is regarded as an effective tool for recognizing pilot fatigue, the direct application of deep learning models to raw EEG signals faces significant challenges due to issues such as massive data volume, excessively long training time, and model overfitting. Moreover, existing feature-based methods often suffer from data redundancy due to the lack of effective feature and channel selections, which compromises the model’s recognition efficiency and accuracy. To address these issues, this paper proposes a framework, named ASFT-Transformer, for fast and accurate detection of pilot fatigue. This framework first extracts time-domain and frequency-domain features from the four EEG frequency bands. Subsequently, it introduces a feature and channel selection strategy based on one-way analysis of variance and support vector machine (ANOVA-SVM) to identify the most fatigue-relevant features and pivotal EEG channels. Finally, the FT-Transformer (Feature Tokenizer + Transformer) model is employed for classification based on the selected features, transforming the fatigue recognition problem into a tabular data classification task. EEG data is collected from 32 pilots before and after actual simulator training to validate the proposed method. The results show that ASFT-Transformer achieved average accuracies of 97.24% and 87.72% based on cross-clip data partitioning and cross-subject data partitioning, which were significantly superior to several mainstream machine learning and deep learning models. Under the two types of cross-validation, the proposed feature and channel selection strategy not only improved the average accuracy by 2.45% and 8.07%, respectively, but also drastically reduced the average training time from above 1 h to under 10 min. This study offers civil aviation authorities and airline operators a tool to manage pilot fatigue objectively and effectively, thereby contributing to flight safety.

## 1. Introduction

Pilot fatigue has been widely acknowledged as a significant safety hazard that systematically impairs cognitive and operational performance, posing a substantial risk for aviation incidents and accidents [1]. Researchers have identified several factors that can be attributed to fatigue, such as sleep loss, high workloads, and circadian misalignment [2,3,4,5]. However, fatigue cannot be eliminated, as the human brain and body are designed to function optimally with unrestricted nightly sleep. Therefore, the focus must shift to its proactive management [1].

As the global guiding framework for fatigue management, International Civil Aviation Organization (ICAO) Doc 9966, “Guidance Manual on Fatigue Management Procedures” [1], with its scientific principles and methodology, serves as an essential foundation for individual states to establish their specific regulations. The Federal Aviation Administration (FAA) [6] of the United States, the European Union Aviation Safety Agency (EASA) [7], and the Civil Aviation Administration of China (CAAC) [8] all referred to this document and issued their own relevant regulations and guidance materials to manage the fatigue risks of pilots. These regulations require airline operators to establish a robust Safety Management System (SMS), which includes setting explicit limits on flight and duty times, ensuring adequate rest periods, and conducting training on pilot fatigue management. Concurrently, they provide for a performance-based and data-driven Fatigue Risk Management System (FRMS) approach, enabling the effective management of fatigue risks through continuous monitoring, data analysis, and risk assessment. Notably, the FRMS approach proposes the use of scales for pilots to self-report their fatigue levels, such as the Stanford Sleepiness Scale (SSS) [9] and the Karolinska Sleepiness Scale (KSS) [10]. However, scale-based self-reporting is highly subjective, potentially yielding unreliable data. For one, the scales categorize fatigue into many levels (e.g., the KSS has 9), which can lead to pilots making inaccurate self-assessments. More seriously, a pilot might report being alert to continue with flight duties or simulator training—despite being fatigued—out of fear of negative performance reviews (such as not logging enough flight hours), which would pose a grave threat to aviation safety. Therefore, the FRMS also recommends the objective analysis of pilot fatigue through the collection and analysis of physiological data to further establish fatigue risk indicators.

The employment of physiological detection techniques, such as electroencephalography (EEG) [11], electrocardiography (ECG) [12], and electromyography (EMG) [13], to assess the fatigue status of aircraft pilots and car drivers has been proven to be objective and practical. Of these, EEG is often considered the most promising method for workload and fatigue assessment [14,15,16,17], as it offers a direct measurement of brain activity [18,19]. And for the purposes of analysis and extracting features, EEG signals are typically divided into several frequency bands: δ band (0.5–4 Hz), θ band (4–8 Hz), α band (8–13 Hz), and β band (13–30 Hz) [20]. These bands are usually used to represent different brain activities; for example, α power is associated with arousal, resource allocation, or workload [17]; frontal θ power is associated with training and performing various flight tasks [21]; and the β/(α+β) energy ratio is one of the most commonly used and studied neurophysiological metrics in aviation research [11]. Additionally, Jing et al. [22] observed that as driving fatigue intensified, there was a corresponding reduction in the amplitude, absolute energy, and average power of the α and β bands. Conversely, these same metrics showed an increase within the θ band.

Nevertheless, accurately identifying the fatigue state using EEG signals remains a challenging task, as such signals are often very weak, have a poor signal-to-noise ratio, and frequently exhibit nonstationary and nonlinear characteristics. Given that deep learning can automatically extract complex EEG features without requiring prior knowledge [23,24,25], multiple artificial neural networks have been proposed as an end-to-end solution for monitoring human functional states. The Convolutional Neural Network (CNN) is widely applied in EEG-based cognitive workload [26], emotion recognition [27], and fatigue detection [28,29,30], demonstrating its effectiveness in these applications. Similarly, the Recurrent Neural Network (RNN) is also gaining popularity for processing EEG data, which is typical sequential data. Tang et al. [31] employed the Long Short-Term Memory (LSTM) to improve the classification performance of fatigue states. Cao et al. [32] achieved precise identification of classroom fatigue by using LSTM and attention mechanisms. Mehmood et al. [33] found that Bi-LSTM achieves superior performance compared to LSTM and 1D-CNN for the task of classifying mental fatigue in construction equipment operators. However, the RNN is less effective at extracting spatial features from EEG signals. Therefore, some studies have focused on improving network structures to enhance feature extraction in both temporal and spatial domains. Pan et al. [34] developed a hybrid CNN-LSTM deep neural network for continuously estimating alertness levels in high-speed train drivers, yielding favorable outcomes. Wang et al. [17] proposed the LGNet for assessing subjects’ cognitive workload based on EEG during simulated flight, achieving an average classification accuracy of 83.26% based on cross-session data partitioning. Similarly, Jia et al. [35] developed a temporal and graph convolution-based (MATCN-GT) fatigue driving detection model, which achieved a 3.25% improvement in accuracy compared to the traditional graph convolutional neural network.

Although using deep learning for end-to-end fatigue detection appears to yield promising results, it is highly demanding in terms of hardware and training time for models, as the volume of raw EEG data is substantial. For instance, in the widely used SEED-VIG public dataset [36], researchers collected EEG data from 23 subjects using equipment with 17 electrode channels, and each electrode channel recorded approximately 2 h of data for each person at a sampling rate of 1000 Hz. These studies have not reported the time required for model training, but it is undoubtedly an extremely time-consuming process. Furthermore, using such multi-dimensional and massive data as input for a neural network is prone to issues such as gradient vanishing, gradient exploding, and model overfitting. Therefore, extracting the appropriate features to reduce complexity can effectively improve the fatigue recognition efficiency [18]. Gao et al. [37] calculated the differential entropy (DE) for five frequency bands in 8 s intervals and used it as input for their constructed CSF-GTNet, achieving a fatigue recognition accuracy of 81.48% on the SEED-VIG dataset. Subsequently, they [38] proposed the SFT-Net, improving the accuracy to 87.13% with the same data. Luo et al. [15] proposed the ASF algorithm to extract three entropy features from EEG data, which has been proven effective in detecting fatigue driving. Additionally, various time-domain and frequency-domain features have been widely utilized as inputs for fatigue state recognition models [16,18,39,40,41]. Some studies have also utilized features from the EEG-based brain functional network [42,43,44]. However, the vast majority of these studies do not conduct feature selection and fusion, which means the volume of data for model training remains extensive. This is because while feature extraction reduces the data’s length, it also increases its dimensionality. Moreover, the same feature is not necessarily effective in characterizing fatigue across different electrode channels and frequency bands, as it may not exhibit a significant change between the fatigue and alert states, potentially impacting the model’s recognition performance [45]. Although Zhou et al. [42] systematically tested 26 feature sets against eight machine learning algorithms to ultimately find the optimal combination of features and algorithms for fatigue detection, this process was lengthy and did not reduce the dimensionality of the data. Liu et al. [39] selected eight optimal electrode channels, then extracted and fused features from them; however, there was ultimately no significant improvement in fatigue detection accuracy for several test subjects. Zhang et al. [18] proposed a method for selecting and fusing the optimal feature subset; however, none of the eight classification models they used achieved an accuracy higher than 80%. Consequently, for EEG-based fatigue state recognition, both feature selection and classification model construction are of crucial importance.

This research aims to develop an accurate and rapid EEG-based method for detecting pilot fatigue, thereby assisting airline operators in managing pilot fatigue. Unlike most of the existing studies, pilots’ EEG signals are not collected during flight. This is because EEG equipment is, to some extent, invasive and can affect pilot cognition and behavior, making it unrealistic for them to wear it during actual flight operations or simulator training. Instead, the pilots’ EEG data is recorded before and after actual simulator training to be used for training the fatigue detection model. In other words, the purpose of this research is to use EEG data to objectively determine whether a pilot is fatigued before flight, thereby enhancing flight safety further.

In summary, combining the current research landscape and practical application needs, this paper proposes a framework named ASFT-Transformer. Based on this framework, the features most correlated with fatigue are input into the classification model to achieve fast and accurate pilot fatigue detection after extracting relevant features from the EEG signals of each electrode channel. The main contributions of this study are as follows:

(1) EEG feature extraction. The EEG signals were initially decomposed into δ, θ, α, and β bands, followed by the extraction of both time-domain and frequency-domain features. This process yielded a multi-dimensional description of the raw signals. Consequently, it improved the interpretability of the features and ensured informational completeness, providing crucial insights for a detailed study of how EEG signals correlate with pilot fatigue.
(2) Pivotal feature and channel selection. A channel and feature selection method based on one-way analysis of variance and support vector machine (ANOVA-SVM) was proposed to select the channels and features closely related to pilot fatigue, thereby preventing the classifier from using excessive irrelevant information that may lead to lower accuracy. Furthermore, this process reduced the data dimensionality, which significantly shortened the model training time.
(3) Classification recognition. The FT-Transformer (Feature Tokenizer + Transformer) was employed to identify pilot fatigue states. For model training, pivotal features from each time window were aggregated into a feature vector, with each vector serving as an independent sample of the pilot state. These vectors were then fed into the classifier for training. Based on the above steps, the fast detection of pilot fatigue has been achieved.

## 2. Methodology

### 2.1. Overall Framework

**Figure 1:**
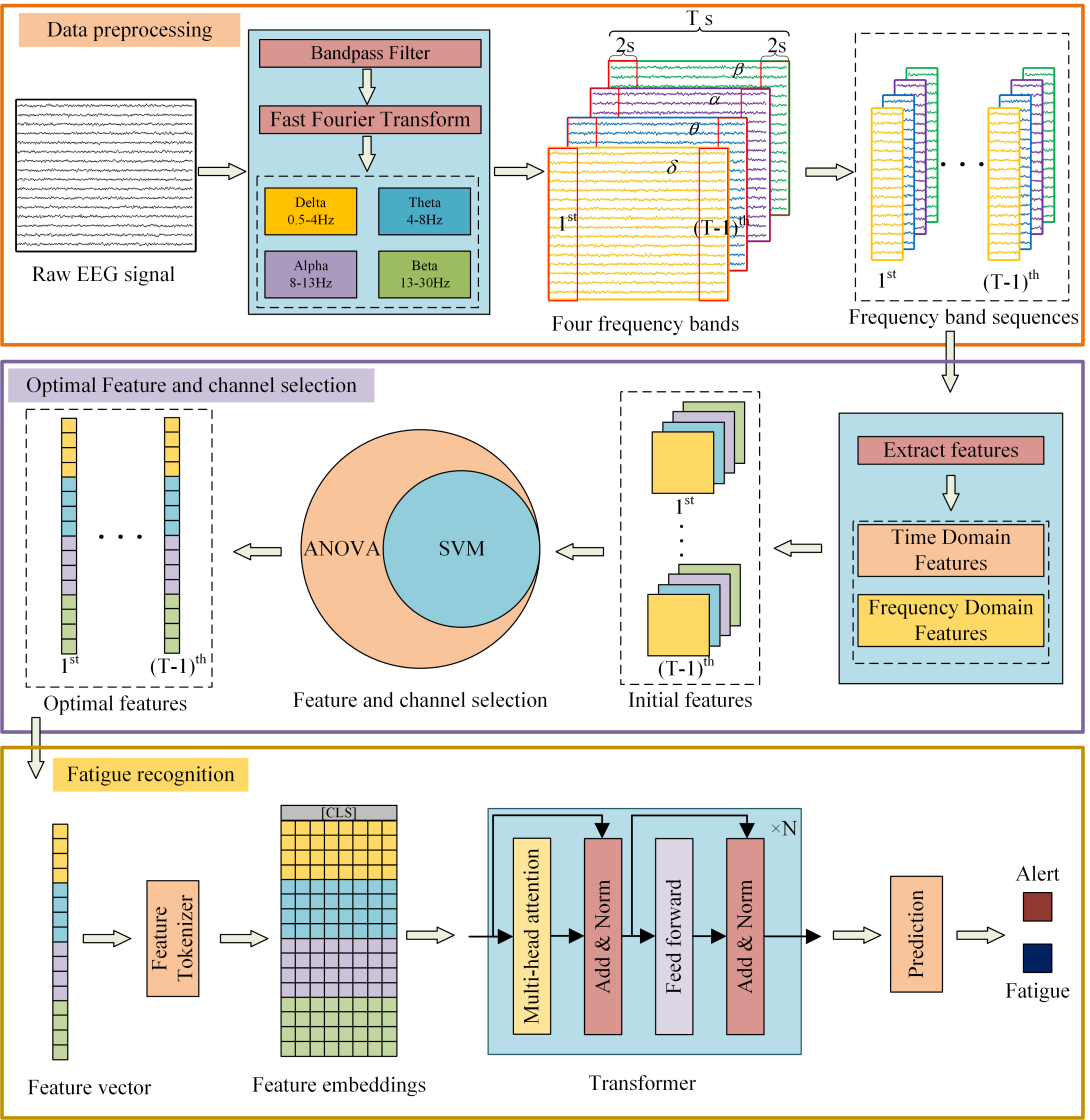
Illustration of the proposed ASFT-Transformer.

Figure 1 illustrates the overall framework of ASFT-Transformer, comprising three key components: data preprocessing, pivotal feature selection, and pivotal channel selection. The details of each part will be introduced in turn.

### 2.2. Data Preprocessing

**Figure 2:**
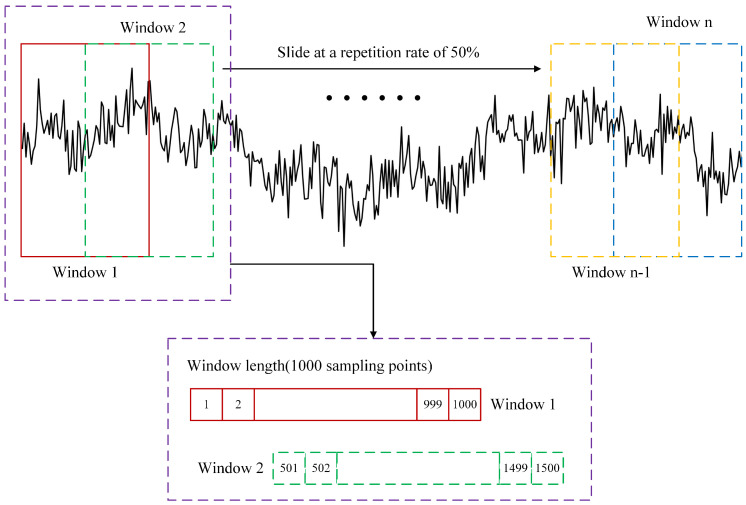
EEG sample interception and decomposition.

A 0.5–30 Hz bandpass filter is first employed to attenuate some artifacts for each single-channel EEG signals, and then the fast Fourier transform (FFT) is used to decompose EEG signals into the δ band (0.5–4 Hz), θ band (4–8 Hz), α band (8–13 Hz), and β band (13–30 Hz). To facilitate feature extraction and weaken the side-lobe interference of signals and smooth the eigenvalues as much as possible, each EEG frequency band sequence is then divided into multiple segments of equal length. Specifically, under the sampling rate (500 Hz) and sampling time (10 min) specified in Section 3.1, each original frequency band sequence consisting of 30,000 sampling points is divided into multiple 2 s signal segments (1000 sampling points), with a 50% overlap between adjacent segments, resulting in a total of 599 signal segments. The signal-sample decomposition and interception are shown in Figure 2.

### 2.3. Feature Extraction

Considering the established effectiveness of both time-domain and frequency-domain features for fatigue detection, features from both domains are extracted from each decomposed EEG signal segment. Based on these studies [16,18,39,40,41], 4 time-domain features and 4 frequency-domain features are extracted from each EEG signal segment, and the specific calculation processes of these features are as described in the following text.

#### 2.3.1. Time-Domain Features

Time-domain features are quantitative metrics derived directly from a signal’s temporal waveform and characterize its macroscopic statistical properties. The mean (MEA) $\bar{X}$, energy (ENE) $X_{e}$, variance (VAR) $X_{var}$, and root mean square (RMS) $X_{rms}$ are extracted from the raw time series *s*, and their mathematical formulations are provided below:

(1) $$\bar{X}=\frac{1}{N}\sum_{i=1}^{N}x_{i}$$

(2) $$X_{e}=\sum_{i=1}^{N}|x_{i}^{2}|$$

(3) $$X_{var}=\frac{1}{N}\sum_{i=1}^{N}{\left(|x_{i}^{2}|-\bar{X}\right)}^{2}$$

(4) $$X_{rms}=\sqrt{\frac{1}{N}\sum_{i=1}^{N}x_{i}^{2}}$$

where *N* is the number of samples, i=1,2,3,…,N and $x_{i}$ is the value of the signal sample *s* at the ith sampling point.

#### 2.3.2. Frequency-Domain Features

Frequency-domain analysis reveals how a signal’s energy is distributed across various frequency bands via its spectrum. Its primary advantage over time-domain analysis lies in its ability to detect subtle signal changes by monitoring shifts in the power of specific frequency bands. Therefore, this method is widely regarded as a more powerful and revealing tool for signal analysis. In the actual frequency-domain analysis of the EEG signal, the fast Fourier transform (FFT) is usually employed to decompose the raw time series *s* [46], thereby further revealing the distribution patterns of the signal at different frequencies. The FFT can be described as

(5) $$S\left(k\right)=\left\{\begin{array}{c} \sum_{N=0}^{L-1}s\left(N\right)\cdot {\left[\cos(2\pi /L\;)-j\sin(2\pi /L\;)\right]}^{kN},0\leq k\leq L-1\\ 0,others \end{array}\right.$$

where *k* is the series number of the EEG frequency domain, *N* is the length of the signal sample *s*, and $L\approx N^{2}$.

Then, the power spectrum density (PSD) $F_{psd}$, centroid frequency (CF) $F_{cf}$, frequency variance (FV) $F_{fv}$, and mean square frequency (MSF) $F_{msf}$ are extracted from the raw time series *s*, and their mathematical formulations are provided below:

(6) $$F_{psd}=\frac{1}{f_{m}}{\sum_{k\in f_{m}}∥S(k)∥}^{2}$$

(7) $$F_{cf}=\frac{\int_{0}^{+\infty }s\cdot S(k)dk}{\int_{0}^{+\infty }S(k)dk}$$

(8) $$F_{fv}=\frac{\int_{0}^{+\infty }\left(s-F_{fc}\right)\cdot S\left(k\right)dk}{\int_{0}^{+\infty }S(k)dk}$$

(9) $$F_{msf}=\frac{\int_{0}^{+\infty }s^{2}\cdot S\left(k\right)dk}{\int_{0}^{+\infty }S(k)dk}$$

where *m* indicates a certain band (δ, θ, α, or β), and $f_{m}$ represents the frequency range of the *m* band.

### 2.4. Pivotal Feature Screening and Channel Selection

Compared to raw EEG signals, extracted multi-dimensional features allow a model to capture more comprehensive information [47]. However, as previously stated, while feature extraction reduces the length of the data, it simultaneously increases its dimensionality. Due to the similarity and correlation between features from adjacent EEG channels, applying both feature screening and channel selection is an effective way to prevent data redundancy and improve the model’s computational efficiency.

#### 2.4.1. Feature Screening

Traditional machine learning models often operate on the assumption that all features within a set are equally important. In practice, however, these feature sets frequently contain irrelevant or redundant information. Consequently, assigning uniform importance to both non-informative and significant features can substantially impair the model’s performance on unseen data [47]. In this study, the one-way analysis of variance (ANOVA) is proposed to select features with statistically significant differences between the alert and fatigue states (*p* < 0.01). Through hypothesis testing, one-way ANOVA directly quantifies the significance level of the mean difference for each feature across different states, yielding results that are highly interpretable, which has been shown to be valid in previous studies related to EEG [18].

#### 2.4.2. Channel Selection

Channel selection aims to identify the most pivotal EEG channels by quantitatively evaluating their correlation with fatigue, thereby not only accelerating fatigue recognition speed but also simplifying the overall experimental scheme. In this study, we propose a method that utilizes the support vector machine (SVM) as a base classifier and the Area Under the Curve (AUC) of the Receiver Operating Characteristic (ROC) curve as the performance metric to quantify each channel’s relevance to fatigue, as the AUC is a widely adopted metric for evaluating the performance of recognition models [48,49], and SVM has been proven to be effective in EEG-based fatigue detection [18,42,43,50]. The specific procedure is as follows:

(1) Individual SVM classifier training

A separate SVM classifier is trained for each of the 16 channels, using pivotal features extracted from the 4 frequency bands as input. The purpose of each SVM model is to distinguish between the ‘alert’ and ‘fatigue’ states based on the information from that single channel.

SVM is a robust supervised learning classifier whose fundamental principle is to find an optimal hyperplane in a high-dimensional feature space that maximally separates data points of different classes. The core idea is to maximize the margin, which is the distance between the separating hyperplane and the nearest data points (the support vectors) from either class. The hyperplane defines the decision boundary of an SVM:

(10) w·x+b=0

where w represents the weight vector perpendicular to the hyperplane, x is the input feature vector, and *b* is the bias term.

To handle data that may not be perfectly linearly separable, a soft-margin SVM is used. Its objective is to find the optimal w and *b* by solving the following convex quadratic programming problem:

(11) $$\min_{w,b,\xi }\;\frac{1}{2}{∥w∥}^{2}+C\sum_{i=1}^{N}\xi_{i}$$

subject to the constraints

(12) $$y_{i}\left(w\cdot x_{i}+b\right)\geq 1-\xi_{i},\xi_{i}\geq 0$$

where *N* is the number of feature vectors, i=1,2,3,…,N, $x_{i}$ is the ith feature vector, $y_{i}\in \left\{-1,1\right\}$ is the fatigue state label, *C* is a regularization parameter that controls the trade-off between maximizing the margin and minimizing the classification error, and $\xi_{i}$ are slack variables that permit some degree of misclassification.

Each EEG channel in each frequency band has an EEG feature dataset $W=\left\{x_{i},y_{i}\right\}$ composed of pivotal features. For each sample $x_{i}$, the SVM model calculates a score based on its signed distance to the hyperplane, given by the decision function f(x)=w·x+b. A positive score classifies the sample as one class, and a negative score as the other, with the magnitude indicating the confidence of the classification.

(2) Evaluating performance with AUC

After training, the performance of each channel’s SVM model is evaluated by calculating its AUC score. The AUC value, ranging from 0 to 1, represents the probability that the classifier will rank a positive sample (alert) higher than a negative sample (fatigue). A higher AUC indicates that the channel provides more discriminative information for fatigue detection. The AUC is calculated as [18]

(13) $$AUC=\frac{\sum_{i\in \mathrm{PositiveClass}}rank_{i}-\frac{T(1+F)}{2}}{T\times F}$$

where *T* is the number of positive samples, *F* is the number of negative samples, and $rank_{i}$ represents the ranking number of the ith positive sample features.

(3) Ranking and selecting pivotal channels

Finally, the EEG channels are ranked based on their resulting AUC scores. Channels that consistently achieve higher AUC values are identified as pivotal for fatigue recognition and are selected for the final classification model, while channels with lower AUC values are discarded.

This systematic process ensures that only the most informative channels are used in the subsequent fatigue recognition step, thereby reducing data redundancy and improving computational efficiency.

### 2.5. Fatigue Recognition Based on EEG Features

**Figure 3:**
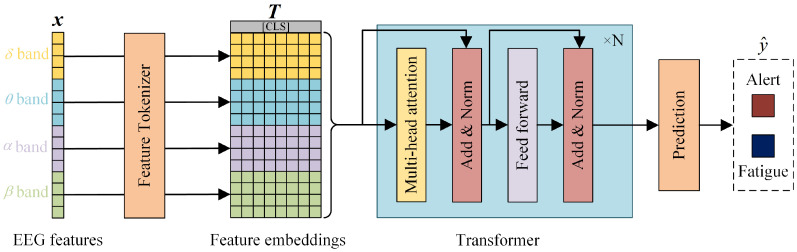
The schematic diagram of the FT-Transformer architecture.

The FT-Transformer (Feature Tokenizer + Transformer) is employed for EEG feature-based pilot fatigue recognition, which is a simple adaptation of the Transformer architecture [51] and has achieved superior prediction performance in a variety of tabular data analysis tasks [52]. Figure 3 briefly demonstrates the main parts of FT-Transformer and its process for recognizing fatigue status using EEG features. In brief, the model transforms all features to embeddings and applies a stack of Transformer layers to the embeddings for further classification.

Specifically, the Feature Tokenizer module transforms the input features x into embeddings $T\in R^{k\times d}$. The embedding for a given feature $x_{j}$ is computed as follows:

(14) $$T_{j}=b_{j}+f_{j}\left(x_{j}\right)\in R^{d}$$

where $b_{j}$ is the jth feature bias. For different types of features (categorical and numerical), the operational process of $f_{j}$ varies; see the original paper [52] for details. Overall,

(15) $$T=stack\left[T_{1},T_{2},\ldots ,T_{k}\right]\in R^{d}$$

For the Transformer model, the embedding of the [CLS] token (or “classification token”, or “output token”) [53] is appended to T, and *L* Transformer layers $F_{1},F_{2},\ldots ,F_{L}$ are applied:

(16) $$T_{0}=stack\left[[CLS],T\right]$$

(17) $$T_{i}=F_{i}\left(T_{i-1}\right)$$

The PreNorm variant is used for easier optimization [54]. In the PreNorm setting, it is necessary to remove the first normalization from the first Transformer layer to achieve good performance. See the original paper [51] for the background on Multi-Head Self-Attention (MHSA) and the Feed-Forward module. See the Supplementary Material for details, including activations, normalization placement, and dropout module placement [55].

The final representation of the [CLS] token is used for prediction:

(18) $$\hat{y}=Linear\left(ReLU\left(LayerNorm\left(T_{L}^{\left[CLS\right]}\right)\right)\right)$$

FT-Transformer stands as a powerful and promising tool for tabular data modeling, achieving state-of-the-art performance on many tasks. Previous studies have also demonstrated the advantages of the Transformer in the analysis of pilots’ EEG signals [56]. Incorporating the Transformer architecture significantly enhances the model’s ability to automatically learn and leverage complex interactions between features. Although it has high requirements for computational resources and data volume, FT-Transformer is undoubtedly a desirable option when dealing with larger datasets where feature dimensions are not high, but their interactions are complex.

## 3. Results

### 3.1. Dataset Description

#### 3.1.1. Participants

A total of 32 male pilots from a certain airline in China participated in the experiment (16 captains and 16 first officers, age: 36.5 ± 9.0). All participants were confirmed to be free of any mental disorders. For the 48 h before the experiment, they were instructed to maintain at least 8 h of sleep per night and to abstain from alcohol, caffeine, and drugs that cause drowsiness. Furthermore, all participants were briefed on the specific experimental procedure before the experiment began, and written informed consent was obtained from each. This study was reviewed by the College of General Aviation and Flight, Nanjing University of Aeronautics and Astronautics, and was found to be in accordance with the ethical principles of the Declaration of Helsinki.

#### 3.1.2. Procedure

**Figure 4:**
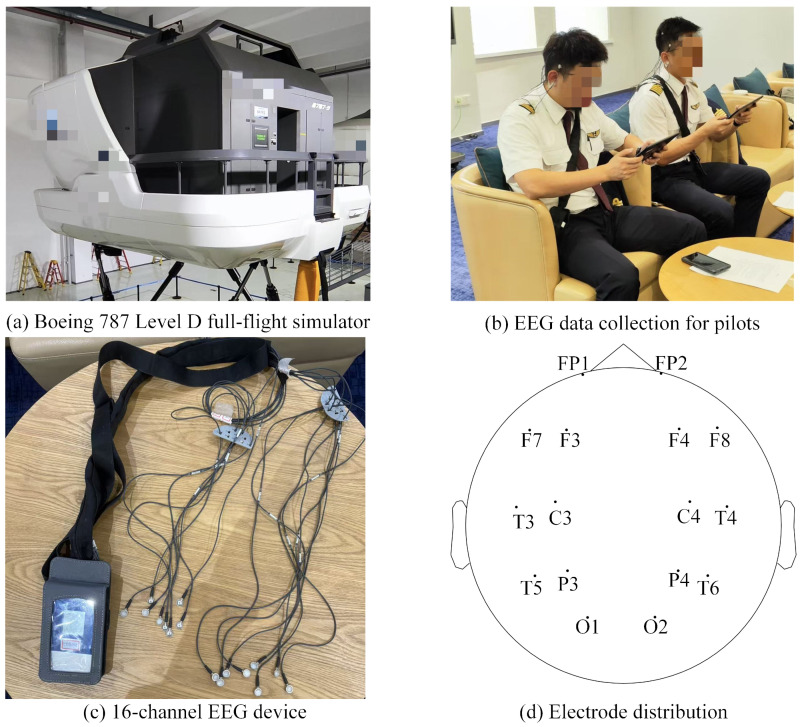
Schematic diagram of experimental equipment.

As previously described, the pilots’ EEG signals were recorded before and after their actual simulator training. Therefore, the experiment consisted of the pilots’ daily simulator training, which was conducted at the airline’s Flight Training Center using a Boeing 787 Level D full-flight simulator, as shown in Figure 4a.

**Figure 5:**
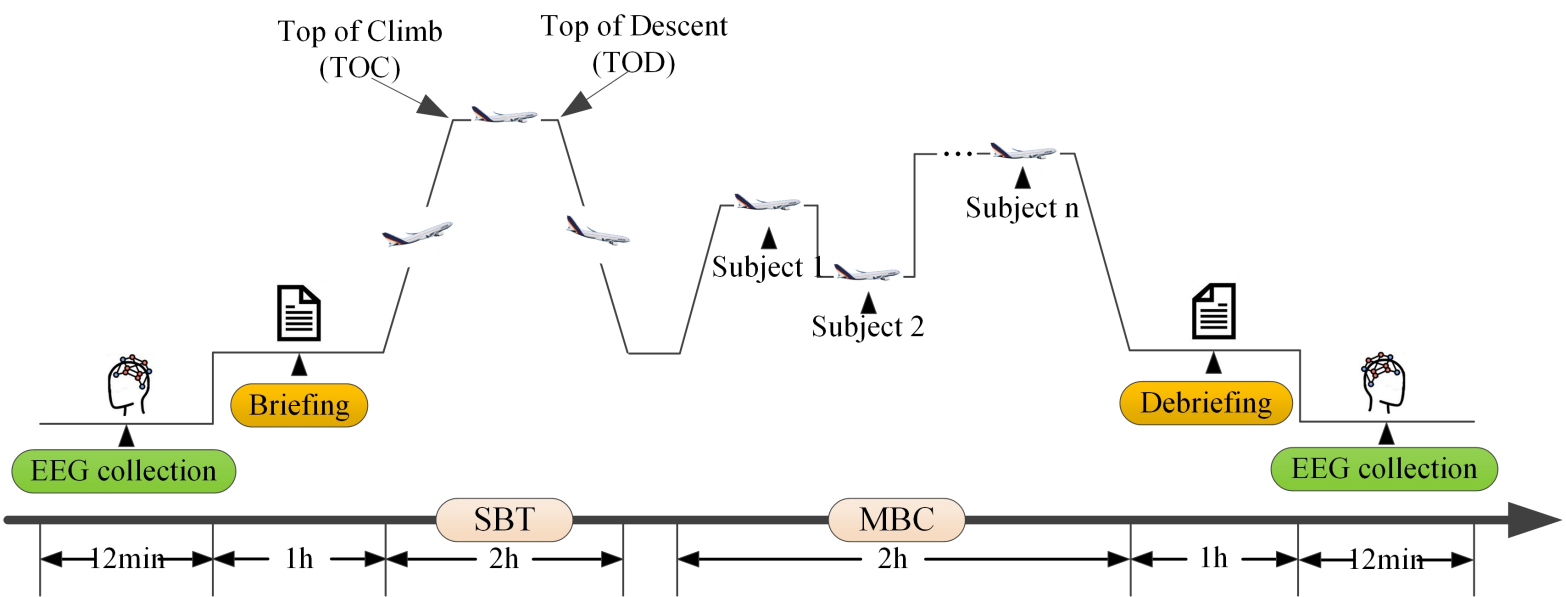
The experimental procedure of Flight Simulator Training and EEG Data Collection for Pilots.

Based on the principles of Evidence-Based Training (EBT) [57], a pilot’s simulator training procedure primarily consists of the following components: 1 h of pre-flight briefing, 2 h of Scenario-Based Training (SBT), 2 h of Maneuver-Based Checks (MBC), and 1 h of pre-flight evaluation and debriefing. Each simulator training procedure involves one captain and one first officer, who are jointly instructed and evaluated by a flight instructor. During the pre-flight briefing, the instructor briefs the crew on the training scenario, the items of the MBC, and the scoring criteria, while also summarizing their past training deficiencies to increase the training efficiency. The SBT consists of the flight crew conducting 1–2 complete flights. These scenarios incorporate a variety of anticipated and unanticipated complications, such as wind shear, thunderstorms, and engine failure. The MBC consists of several different subjects, such as a rejected takeoff, stall recovery, and landing with crosswind. During the pre-flight evaluation and debriefing phase, the instructor provides fair and objective feedback based on the crew’s observed actions and factual information. The goal is to ensure that the crew members clearly understand their performance and can identify areas for improvement. The entire experimental procedure is shown in Figure 5.

#### 3.1.3. Data Acquisition

Delica AEEG-3202 (Shenzhen Delica Medical Equipment Co., Ltd., Shenzhen, China), a 16-channel electroencephalograph, was employed to collect pilots’ EEG signals before and after the simulator training, as shown in Figure 4b. The system utilized standard Ag/AgCl disc-type wet electrodes to ensure high-fidelity signal acquisition with a high signal-to-noise ratio, and all signals were recorded at a sampling rate of 500 Hz. The EEG instrument consists of 16 EEG electrodes and a small control box, as shown in Figure 4c, and Figure 4d depicts the electrode distribution for these channels. Regarding the electrode names, the letters represent regions of the cerebral cortex: FP stands for Frontopolar, F for Frontal, C for Central, P for Parietal, T for Temporal, and O for Occipital. The numbers indicate the position on the left or right hemisphere: odd numbers (1, 3, 5, 7) represent the left hemisphere, and even numbers (2, 4, 6, 8) represent the right hemisphere. The smaller the number, the closer the electrode is to the midline of the head.

Since this study aims to detect pilot fatigue quickly based on EEG signals and to minimize the impact on the pilot state through prolonged EEG device wear, only approximately 12 min of EEG data was collected from each pilot before and after their training. Based on findings from previous research [1,2,3,4,5,6,7,8], it is established that pilots enter a fatigued state after 6h of professional flight training. Therefore, the pretraining EEG data were defined as the ‘alertness state’ data, while the pretraining EEG data were defined as the ‘fatigue state’ data. And to balance the dataset, only the first 10 min of each recording was subsequently used.

### 3.2. Selection of Significant Features

To comprehensively identify the features most relevant to pilot fatigue, the one-way ANOVA was used to determine whether the original eight features (as described in Section 2.3) showed statistically significant differences between the ’alert’ and ’fatigue’ states at a 99% confidence level (*p* < 0.01) within a certain frequency band and channel. The detailed statistical results for each feature across all channels and bands are presented in Table A1, Table A2, Table A3 and Table A4 (Appendix A).

The results indicated that the mean (MEA) feature showed no statistical correlation with pilot fatigue in any frequency band, with *p*-values consistently greater than 0.01, which confirms the discovery of Zhang et al. [18]. Similarly, the frequency variance (FV) feature showed statistically significant changes in fewer than 10 channel-band combinations, suggesting it is not a reliable indicator. In contrast, the remaining six features exhibited statistically significant changes in over 40 band–channel combinations. Notably, within the beta band, both CF and MSF values showed significant changes across all channels. Based on these findings, the MEA and FV features were discarded, and the remaining six features were retained as the pivotal feature subset for the subsequent channel selection and classification tasks, as they were determined to be highly relevant to pilot fatigue. The effectiveness of this process is demonstrated in Section 3.4.3.

### 3.3. AUC Calculation for EEG Channels

As described in Section 2.4.2, the purpose of calculating the AUC for each channel was to establish a quantitative metric for evaluating and comparing each channel’s individual ability to distinguish between the ‘alert’ and ‘fatigue’ states. A higher AUC score signifies a stronger correlation with fatigue, marking the channel as a more informative candidate for the final recognition model. For each of the 16 channels, an SVM model was trained using only the six pivotal features of all frequency bands identified in the previous section. The performance of each channel’s model was then quantified by calculating the AUC. To minimize the error, a 10-fold cross-validation strategy was employed, and the final performance metric was reported as the average across all 10 folds.

**Figure 6:**
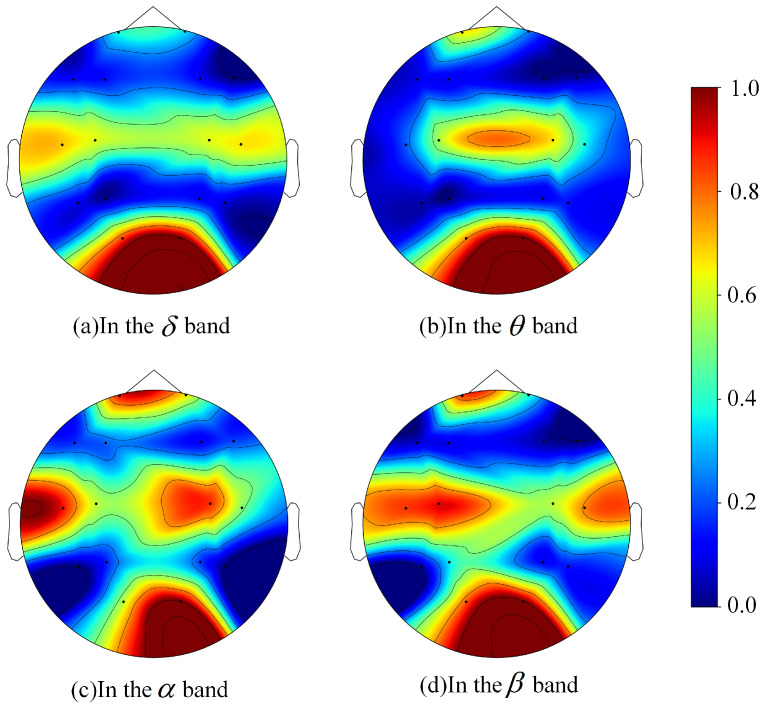
Brain topography of the AUC of each frequency band.

To visualize the correlation between each channel and pilot fatigue, the AUC scores were normalized and plotted on brain topographic maps for each frequency band, as shown in Figure 6. The color transition from blue to red indicates an increasing AUC value, signifying a stronger correlation with fatigue. Across all four frequency bands, channels O1 and O2, located in the occipital region, consistently showed high AUC values, identifying them as pivotal channels for fatigue recognition. This aligns with the findings of Pal et al. [58]. In particular, the O2 channel’s AUC ranked first in each frequency band. In addition, in the α and β frequency bands, the red areas in the central region and parts of the temporal regions are also large, indicating that there may be potential pivotal channels for pilot fatigue in these regions. In contrast, across all frequency bands, the channels located in the frontal region (F3, F4, F7, F8), parietal region (P3, P4), and temporal regions farther from the midline of the head (T5, T6) are blue, indicating that these channels have a weak correlation with fatigue.

Table 1 shows the average AUC of the 16 channels in the four frequency bands (arranged in descending order of size). In particular, regardless of the frequency band, the top eight channels ranked by AUC were invariably the following: O1, O2, T3, T4, C3, C4, FP1, and FP2. In contrast, the average AUC for the other eight channels never exceeded 0.5 in any frequency band. Therefore, the aforementioned channels were selected as the key EEG channels for representing pilot fatigue.

**Figure 7:**
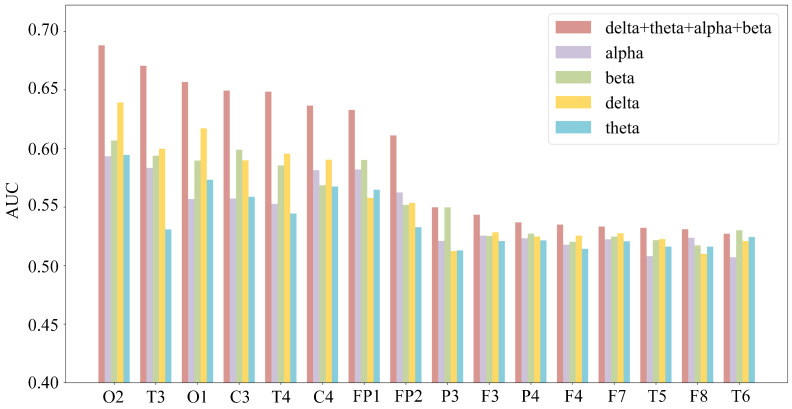
Comparison of the AUC values for SVM classification using different frequency bands’ characteristics for each electrode channel.

Furthermore, although the maximum mean AUC for these channels did not exceed 0.65, which typically indicates poor classification performance, the method of using AUC to select pivotal channels has been proven effective [18]. In practice, for any given channel within a single frequency band, only six features were used for SVM training, indicating that the amount of helpful information available to the model was minimal. To test this hypothesis, a new classification was performed for each channel using a combined set of all six time-domain and frequency-domain features from all frequency bands, and the resulting average AUC was calculated 10 times. As shown in Figure 7, the results confirmed that for the vast majority of these 16 channels, the AUC achieved using features from all bands was higher than that from any single band. Moreover, as will be demonstrated in Section 3.4.3 of this paper, the fatigue recognition accuracy improved significantly after this channel selection process, further supporting our hypothesis.

### 3.4. Pilot Fatigue Recognition

#### 3.4.1. Dataset Description

From the processing steps described in Section 2.2, the collected pilot EEG signals were segmented. Specifically, the 10 min data from each frequency band was segmented into 599 epochs, each with a duration of 2 s and a 50% repetition rate with the next. Following feature extraction and selection, the pivotal features across various dimensions from each 2 s EEG segment constitute a single, independent sample for pilot fatigue recognition. As detailed in Section 3.2 and Section 3.3, a total of six features and eight channels were identified as being highly relevant to pilot fatigue. This resulted in a final feature vector of length 192 for each sample (4 frequency bands × 6 features × 8 channels), yielding a total dataset of 38,336 samples (599 EEG epochs × 32 pilots × 2 sessions).

#### 3.4.2. Model Setup

Our training was performed using Python 3.10 and PyTorch 2.8.0 on a workstation with an Intel(R) Core(TM) i9-10900X CPU and two NVIDIA A5000 GPUs with 64 GB of RAM. Notably, Gorishniy et al. [52] provided a default configuration for the FT-Transformer, which achieved good results on most tasks. However, during our actual model training, it was found that the loss function completely failed to converge when using their recommended settings. Therefore, while preserving the core architecture of the FT-Transformer, a set of lightweight structure parameters better suited to this study’s data scale and computational efficiency was selected, as shown in Table 2. Additionally, the training parameters were set as follows: the learning rate was 0.001, the batch size was 64, and the model was trained for 100 epochs.

All experimental results utilize four commonly used classification metrics: accuracy, precision, recall, and F1_Score. Their calculation formulas are as follows:

(19) $$Accuracy=\frac{TP+TN}{TP+FP+FN+TN}$$

(20) $$Precision=\frac{TP}{TP+FP}$$

(21) $$Recall=\frac{TP}{TP+FN}$$

(22) $$F1\_score=\frac{2\times Precision\times Recall}{Precision+Recall}$$

where *TP* (True Positive) represents the number of positive samples correctly predicted, *TN* (True Negative) represents the number of negative samples correctly predicted, *FP* (False Positive) represents the number of positive samples incorrectly predicted, and *FN* (False Negative) represents the number of negative samples incorrectly predicted.

#### 3.4.3. Recognition Performances

The four-fold cross-validation based on cross-clip data partitioning was first employed to evaluate the performance of our methods; that is, all EEG feature samples were randomly divided into four folds to implement the cross-validation. With the cross-clip data partitioning scheme, the recognition results were first compared under three distinct conditions (Table 3): (1) without using the pivotal feature screening and channel selection methods (FT-Transformer), (2) using only the pivotal feature screening method (AFT-Transformer), and (3) using both the pivotal feature screening and pivotal channel selection methods (ASFT-Transformer).

It is worth noting that the ASFT-Transformer model achieved an average accuracy of 97.24% for pilot fatigue recognition, surpassing the results of the baseline FT-Transformer model (94.79%) and the AFT-Transformer model (95.27%). This indicates that both the pivotal feature screening method and channel selection method proposed in this study can effectively improve the accuracy of pilot fatigue recognition. Furthermore, by selecting the pivotal features and channels, the average training time of the FT-Transformer was significantly reduced from 56 min 32.3 s to 8 min 38.8 s.

Subsequently, our model was benchmarked against several standard deep learning and classic machine learning models for tabular data classification, including ResNet, Multilayer Perceptron (MLP), XGBoost, SVM, Logistic Regression (LR), and k-Nearest Neighbors (KNN). Furthermore, to more comprehensively highlight the advantages of our proposed method, it was also compared with three time-series deep learning models commonly used for EEG-based fatigue detection: LSTM, BiLSTM, and 1D_CNN [59]. To ensure a fair comparison, the input of these three models consisted of the raw data from the four frequency bands. Specifically, as detailed in Section 2.2, an independent sample was defined as a 1000-time-step (2 s) sequence from a single pilot EEG data, with a 50% overlap between consecutive samples. The performance comparison results are shown in Table 4.

Overall, the three deep learning methods, ASFT-Transformer, ResNet, and MLP, all performed well on the task of EEG feature-based pilot fatigue recognition, with average accuracies of 97.24%, 96.51%, and 94.85%, respectively. This also reflects the advantages of deep learning for tabular data classification tasks. Although our method required a little longer training time, it achieved the best fatigue recognition performance, and its average training time is still considered very reasonable. In comparison, most of the classic machine learning models (SVM, LR, and k-NN) performed less impressively. Although the average training times for LR and k-NN were both under 1min, their average accuracies were only slightly above 70%. Notably, the XGBoost model required an average training time of just 37.3 s yet achieved an average fatigue recognition accuracy of 96.03%. The performance of the common time-series deep learning models using raw EEG band data for pilot fatigue recognition was even less ideal. Of the three, the top-performing 1D_CNN merely reached an average accuracy of 76.88%, which is insufficient to meet practical application requirements. And for LSTM and BiLSTM, not only were the average training times very long (approximately 1 h and 2.5 h, respectively), but the classification performance was also very poor (with average accuracies of 57.68% and 58.89%, respectively).

**Figure 8:**
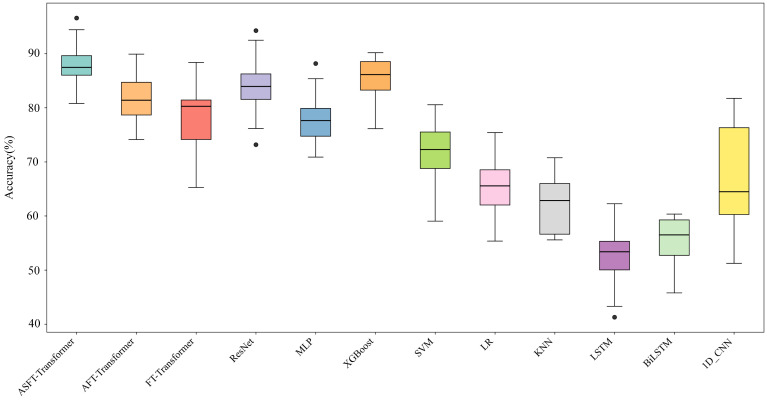
Comparison of classification accuracy of different methods based on cross-subject data partitioning.

To further evaluate the model’s robustness and prevent the information leakage that can occur in cross-clip data partitioning (where a single pilot’s EEG data could appear in both training and validation sets), a five-repetition, four-fold cross-validation based on cross-subject data partitioning was adopted. In this procedure, the 32 pilots were randomly partitioned into four subject-disjoint folds, and a complete four-fold cross-validation was then performed, where each fold was iteratively used for validation while the other three were used for training. This entire process was repeated five times with different random partitions of the subjects to genuinely evaluate the model’s generalization capability on unseen subjects. The overall experimental results are shown in Table 5, and the detailed distribution of classification accuracies is presented in Figure 8 to illustrate the stability and robustness of the models.

Not only do the results show that ASFT-Transformer achieved the highest average and median accuracy, demonstrating superior overall performance, but, more importantly, its exceptionally narrow box and short whiskers are also evidence of its stability and robustness. This indicates that its performance is highly consistent and reliable across different data partitions.

In general, the ASFT-Transformer model achieves accurate and robust recognition of pilot fatigue from EEG features while simultaneously avoiding information redundancy, and its effectiveness has been thoroughly validated in actual experiments.

## 4. Discussion

The pre-flight evaluation of pilot fatigue state using EEG signals is regarded as an objective fatigue management tool with practical significance for enhancing aviation safety. Therefore, this study collected EEG data from 32 pilots before and after a 6 h actual simulator training session to construct a fatigue recognition model. Notably, the main challenge in EEG-based fatigue detection lies in the complexity and instability of raw EEG data. Although previous research indicates that deep learning techniques are effective in addressing these difficulties, the large volume of raw EEG data not only increases the training time for neural networks but can also easily lead to problems such as vanishing gradients, exploding gradients, and overfitting. At least in our specific experiments, using raw EEG frequency band data to recognize pilot fatigue did not yield good results (Table 4 and Table 5). To reduce the model’s input while ensuring it could capture comprehensive information about fatigue, multi-dimensional features were extracted from the EEG signal segments (2 s). The pilot fatigue recognition problem is thereby transformed into a tabular data classification task.

The ASFT-Transformer model was designed to classify fatigue states based on EEG features, thereby avoiding data redundancy and improving the model’s computational efficiency. The ANOVA-SVM model was first proposed for pivotal feature screening and channel selection. Specifically, the ANOVA was used to select six time-domain and frequency-domain features that showed a statistically significant difference (*p* < 0.01) under alertness and fatigue (Table A1, Table A2, Table A3 and Table A4). And the AUC value for each channel was then calculated within each of the four frequency bands to assess the relevance of each EEG channel to the fatigue state. This was conducted using the SVM to classify fatigue states based on these six pivotal features. As shown in Table 1, eight key channels were selected to represent pilot fatigue. Subsequently, these features from different dimensions were used to train the FT-Transformer classification model.

To validate the reliability of the proposed pivotal feature screening and channel selection method in this study, the performance of the FT-Transformer model was tested under three conditions: without feature or channel selection, with only feature selection, and with both feature and channel selection. The results (Table 3 and Table 5) show that both feature and channel selection can improve the model’s recognition accuracy. ASFT-Transformer achieved an average accuracy of 97.24% based on cross-clip data partitioning and an average accuracy of 87.72% based on cross-subject data partitioning, higher than those of the AFT-Transformer model and the FT-Transformer model. Additionally, by selecting the pivotal features and channels, the feature vector length for each sample was reduced from an initial 512 to 192, which significantly decreases the input dimensionality of the data and enhances the model’s computational efficiency, and the average training time of the FT-Transformer was reduced from 56min32.3s to 8min38.8s, making it more suitable for practical applications.

To further validate the effectiveness of the FT-Transformer in recognizing pilot fatigue based on EEG features, it has been compared with several mainstream models for tabular data classification. In different cross-validation methods (Table 4 and Table 5), the FT-Transformer achieves the highest average accuracy, precision, recall, and F1_Scores on the same dataset. Its performance is significantly superior to that of other machine learning and deep learning methods, demonstrating that it can effectively mine the complex, nonlinear relationships among high-dimensional EEG features through its unique feature tokenization and powerful self-attention mechanism, achieving a more accurate and robust classification of pilot fatigue state. In particular, it was noted that the XGBoost model achieved an average accuracy of 96.03% based on cross-clip data partitioning and an average accuracy of 84.02% based on cross-subject data partitioning, with an average training time of less than 1min, which suggests that it could, to some extent, be considered a viable alternative method. Additionally, it has been found that in many EEG studies related to fatigue and workload, independent samples are typically derived from 8 s [36,37,38,40,59], or even more [33,60], of EEG data. In contrast, each of our samples originates from only 2 s of EEG signals, which means the method proposed in this paper utilizes less EEG information yet still achieves accurate fatigue detection.

In summary, a fast, accurate, and robust method has been developed for identifying pilot fatigue, exhibiting significant improvement over other techniques. However, there is still room for optimization. In terms of data acquisition, an EEG device with dry electrodes can be used to collect pilot EEG information, which improves user comfort and simplifies the collection process and offers significant advantages for long-term signal monitoring [61]. As to analysis of signals, the number and dimensionality of features could be expanded, for instance, by adding some entropy features [15,37] or brain functional network features [42,43,44] to incorporate more comprehensive information. Building on this, after applying the pivotal feature screening and channel selection method proposed in this paper, an optimal feature subset selection method can be designed to reduce data dimensionality further and improve the classification model’s performance. Furthermore, future research will focus on validating and testing the model on larger and more diverse datasets. The aforementioned improvements are anticipated to further enhance the model’s robustness and generalization ability, enabling its broader application in various practical scenarios.

## 5. Conclusions

This paper proposes a fast and accurate pilot fatigue recognition framework (ASFT-Transformer), which first utilizes ANOVA and SVM to select the highly fatigue-related EEG features and electrode channels, respectively. The FT-Transformer then projects the multi-dimensional EEG features into high-dimensional embeddings via the Feature Tokenizer and subsequently employs a multilayer Transformer Encoder’s self-attention mechanism to learn the deep interaction relationships among these feature embeddings. Finally, the model aggregates this global information through the classification token ([CLS]) to achieve a classification of pilot fatigue state. Based on cross-clip data partitioning and cross-subject data partitioning, ASFT-Transformer achieved average accuracies of 97.24% and 87.72%, respectively, achieving the highest recognition accuracy compared to existing mainstream methods. Under the same cross-validation conditions, its feature and channel selection strategy increased average accuracies by 2.45% and 8.07%, respectively, and decreased the average training time from above 1 h to under 10 min. These experimental results show the proposed method’s effectiveness, reliability, and robustness for pilot fatigue recognition.

This method provides civil aviation authorities and operators with an objective and effective tool for pilot fatigue management. For instance, a fatigue detection system can be built using our proposed framework and reliable pilot EEG data. Such a system would overcome the limitations of subjective assessments by objectively evaluating a pilot’s condition before a flight. Furthermore, grounded in a data-driven philosophy, this method has a positive impact on authorities and operators by helping them improve and optimize fatigue management training programs. This, in turn, assists flight crews in achieving and maintaining prescribed safety performance levels, thereby ensuring the continuous improvement of operational safety standards.

Future research will build upon expanded feature dimensions and datasets by designing a method for optimal feature subset selection. This is intended to enhance the model’s information processing and learning capabilities, thereby improving the robustness of fatigue recognition. Notably, the proposed method exhibits promising transferability, making it applicable to other EEG-based user state assessments, such as workload detection, attention assessment, and emotion recognition.

## Figures and Tables

**Table 1:** The AUC of channels.

δ Band		θ Band		α Band		β Band	
Channels	AUC	Channels	AUC	Channels	AUC	Channels	AUC
**O2**	0.6393 ± 0.0513	**O2**	0.5945 ± 0.0517	**O2**	0.5934 ± 0.0214	**O2**	0.6068 ± 0.0582
**O1**	0.6172 ± 0.0250	**O1**	0.5733 ± 0.0479	**T3**	0.5834 ± 0.0429	**C3**	0.5989 ± 0.0306
**T3**	0.5998 ± 0.0267	**C4**	0.5675 ± 0.0459	**FP1**	0.5821 ± 0.0504	**T3**	0.5938 ± 0.0305
**T4**	0.5956 ± 0.0529	**FP1**	0.5648 ± 0.0312	**C4**	0.5815 ± 0.0246	**FP1**	0.5902 ± 0.0332
**C4**	0.5904 ± 0.0423	**C3**	0.5587 ± 0.0547	**FP2**	0.5625 ± 0.0495	**O1**	0.5897 ± 0.0424
**C3**	0.5899 ± 0.0411	**T4**	0.5445 ± 0.0578	**C3**	0.5573 ± 0.0206	**T4**	0.5855 ± 0.0593
**FP1**	0.5578 ± 0.0363	**FP2**	0.5328 ± 0.0557	**O1**	0.5569 ± 0.0254	**C4**	0.5686 ± 0.0205
**FP2**	0.5535 ± 0.0486	**T3**	0.5309 ± 0.0414	**T4**	0.5527 ± 0.0328	**FP2**	0.5519 ± 0.0319
F3	0.5284 ± 0.0289	T6	0.5244 ± 0.0447	F3	0.5256 ± 0.0525	P3	0.5497 ± 0.0260
F7	0.5276 ± 0.0575	P4	0.5215 ± 0.0282	F8	0.5238 ± 0.0299	T6	0.5302 ± 0.0454
F4	0.5256 ± 0.0342	F3	0.5210 ± 0.0383	P4	0.5234 ± 0.0451	P4	0.5273 ± 0.0262
P4	0.5248 ± 0.0267	F7	0.5208 ± 0.0542	F7	0.5225 ± 0.0353	F3	0.5253 ± 0.0576
T5	0.5226 ± 0.0342	F8	0.5162 ± 0.0349	P3	0.5211 ± 0.0469	F7	0.5247 ± 0.0295
T6	0.5210 ± 0.0548	T5	0.5162 ± 0.0338	F4	0.5178 ± 0.0259	T5	0.5217 ± 0.0318
P3	0.5124 ± 0.0292	F4	0.5143 ± 0.0215	T5	0.5081 ± 0.0542	F4	0.5203 ± 0.0337
F8	0.5101 ± 0.0260	P3	0.5130 ± 0.0383	T6	0.5071 ± 0.0583	F8	0.5172 ± 0.0240

**Table 2:** Comparison of structural parameter settings and default settings of the FT-Transformer.

Parameters	Ours	Gorishniy [52]
Embedding Dimension	64	192
Number of Transformer Encoder Layers	3	6
Number of Attention Heads	8	8
Dimension of the Feed-Forward Network’s Hidden Layer	128	384
Dropout Rate	0.2	0.1

**Table 3:** Comparison of the performance of FT-Transformer under distinct conditions based on cross-clip data partitioning.

Methods	Accuracy	Precision	Recall	F1_Score	Average Training Time
FT-Transformer	94.79%	94.80%	94.78%	94.79%	56 min 32.3 s
	(±0.84%)	(±0.84%)	(±0.85%)	(±0.86%)	
AFT-Transformer	95.27%	95.28%	95.28%	95.29%	21 min 44.7 s
	(±0.52%)	(±0.51%)	(±0.50%)	(±0.52%)	
ASFT-Transformer	**97.24%**	**97.25%**	**97.25%**	**97.24%**	**8 min 38.8 s**
	(±0.27%)	(±0.27%)	(±0.27%)	(±0.29%)	

**Table 4:** Comparison of the performance of different methods based on cross-clip data partitioning.

Methods	Accuracy	Precision	Recall	F1_Score	Average Training Time
**ASFT-Transformer**	**97.24%**	**97.25%**	**97.25%**	**97.24%**	8 min 38.8 s
	(±0.27%)	(±0.27%)	(±0.27%)	(±0.29%)	
ResNet	96.51%	96.51%	96.49%	96.50%	5 min 30.0 s
	(±0.65%)	(±0.70%)	(±0.63%)	(±0.69%)	
MLP	94.85%	94.85%	94.87%	94.87%	2 min 43.4 s
	(±0.31%)	(±0.28%)	(±0.33%)	(±0.29%)	
XGBoost	96.03%	95.99%	96.01%	96.00%	37.3 s
	(±026%)	(±0.27%)	(±0.26%)	(±0.26%)	
SVM	84.15%	84.18%	84.15%	84.13%	7 min 19.3 s
	(±0.42%)	(±0.39%)	(±0.41%)	(±0.40%)	
LR	72.02%	72.03%	72.02%	72.02%	**12.1 s**
	(±0.35%)	(±0.36%)	(±0.35%)	(±0.37%)	
KNN	71.53%	72.44%	71.50%	71.46%	31.5 s
	(±0.45%)	(±0.40%)	(±0.46%)	(±0.48%)	
LSTM	57.68%	57.02%	55.71%	58.73%	106 min 9.1 s
	(±0.53%)	(±1.48%)	(±2.76%)	(±0.93%)	
BiLSTM	58.89%	62.43%	60.14%	59.64%	245 min 30.8 s
	(±0.54%)	(±0.75%)	(±2.30%)	(±1.57%)	
1D_CNN	76.88%	77.42%	80.21%	81.95%	41 min 0.5 s
	(±5.98%)	(±1.32%)	(±7.35%)	(±11.73%)	

**Table 5:** Comparison of the performance of different methods based on cross-subject data partitioning.

Methods	Accuracy	Precision	Recall	F1_Score
**ASFT-Transformer**	**87.72%**	**87.59%**	**91.27%**	**88.94%**
	(±3.76%)	(±4.26%)	(±5.75%)	(±6.60%)
AFT-Transformer	82.93%	83.43%	83.28%	81.34%
	(±5.01%)	(±4.68%)	(±6.10%)	(±6.78%)
FT-Transformer	79.65%	79.56%	81.01%	81.03%
	(±5.51%)	(±5.08%)	(±7.92%)	(±6.79%)
ResNet	84.25%	84.40%	87.68%	86.97%
	(±5.94%)	(±5.81%)	(±7.12%)	(±6.56%)
MLP	78.42%	79.29%	82.37%	80.25%
	(±4.17%)	(±4.23%)	(±6.96%)	(±5.77%)
XGBoost	84.02%	84.86%	89.75%	85.98%
	(±3.65%)	(±3.88%)	(±5.89%)	(±5.07%)
SVM	71.24%	70.62%	75.40%	72.58%
	(±4.45%)	(±4.75%)	(±6.86%)	(±5.52%)
LR	63.82%	63.42%	67.39%	64.39%
	(±4.37%)	(±4.10%)	(±6.26%)	(±4.57%)
KNN	60.61%	60.20%	63.11%	61.94%
	(±4.37%)	(±4.10%)	(±6.26%)	(±4.57%)
LSTM	53.17%	53.28%	60.29%	58.56%
	(±4.96%)	(±7.43%)	(±9.65%)	(±6.28%)
BiLSTM	55.71%	56.38%	62.77%	60.97%
	(±3.91%)	(±3.49%)	(±8.75%)	(±6.98%)
1D_CNN	69.91%	68.40%	75.18%	72.74%
	(±9.84%)	(±5.10%)	(±9.91%)	(±9.80%)

## Data Availability

The original contributions presented in this study are included in the article. Further inquiries can be directed to the corresponding author(s).

## Abbreviations

The following abbreviations are used in this manuscript:

ANOVA	One-Way Analysis of Variance
AUC	Area Under the Curve
CAAC	Civil Aviation Administration of China
EASA	Convolutional Neural Network
EBT	Evidence-Based Training
ECG	Electrocardiography
EEG	Electroencephalography
EMG	Electromyography
FAA	Federal Aviation Administration
FN	False Negative
FP	False Positive
FRMS	Fatigue Risk Management System
FT	Feature Tokenizer
ICAO	International Civil Aviation Organization
KNN	k-Nearest Neighbors
KSS	Karolinska Sleepiness Scale
LSTM	Long Short-Term Memory
LR	Logistic Regression
MBC	Maneuver-Based Checks
MHSA	Multi-Head Self-Attention
MLP	Multilayer Perceptron
RNN	Recurrent Neural Network
ROC	Receiver Operating Characteristic
SBT	Scenario-Based Training
SMS	Safety Management System
SSS	Stanford Sleepiness Scale
SVM	Support Vector Machine
TN	True Negative
TP	True Positive

## Appendix A. ANOVA Statistical Results of Features in Different EEG Frequency Bands Under Alertness and Fatigue

**Table A1:** ANOVA statistical results of features in the δ band.

Channels	Features							
	MEA	ENE	VAR	RMS	PSD	CF	FV	MSF
FP1	0.9724	<0.01 (*)	<0.01 (*)	<0.01 (*)	<0.01 (*)	<0.01 (*)	<0.01 (*)	<0.01 (*)
FP2	0.9779	<0.01 (*)	<0.01 (*)	<0.01 (*)	<0.01 (*)	<0.01 (*)	<0.01 (*)	<0.01 (*)
F3	0.9757	<0.01 (*)	<0.01 (*)	0.8251	<0.01 (*)	<0.01 (*)	0.4220	<0.01 (*)
F4	0.9581	<0.01 (*)	<0.01 (*)	0.7304	<0.01 (*)	0.9579	0.1814	0.6913
C3	0.9953	<0.01 (*)	<0.01 (*)	<0.01 (*)	<0.01 (*)	<0.01 (*)	<0.01 (*)	<0.01 (*)
C4	0.9591	<0.01 (*)	<0.01 (*)	<0.01 (*)	<0.01 (*)	<0.01 (*)	<0.01 (*)	<0.01 (*)
P3	0.9909	0.2070	0.2070	0.4118	0.1923	<0.01 (*)	0.7526	<0.01 (*)
P4	0.9516	0.3404	0.3404	<0.01 (*)	0.3583	<0.01 (*)	0.6766	<0.01 (*)
O1	0.9400	<0.01 (*)	<0.01 (*)	<0.01 (*)	<0.01 (*)	<0.01 (*)	<0.01 (*)	<0.01 (*)
O2	0.9211	<0.01 (*)	<0.01 (*)	<0.01 (*)	<0.01 (*)	<0.01 (*)	<0.01 (*)	<0.01 (*)
F7	0.9893	<0.01 (*)	<0.01 (*)	<0.01 (*)	<0.01 (*)	<0.01 (*)	<0.01 (*)	<0.01 (*)
F8	0.9783	<0.01 (*)	<0.01 (*)	0.0673	<0.01 (*)	0.1320	0.6174	0.1885
T3	0.9754	<0.01 (*)	<0.01 (*)	<0.01 (*)	<0.01 (*)	<0.01 (*)	<0.01 (*)	<0.01 (*)
T4	0.9915	<0.01 (*)	<0.01 (*)	<0.01 (*)	<0.01 (*)	<0.01 (*)	<0.01 (*)	<0.01 (*)
T5	0.9769	<0.01 (*)	<0.01 (*)	<0.01 (*)	<0.01 (*)	<0.01 (*)	0.6768	<0.01 (*)
T6	0.9993	<0.01 (*)	<0.01 (*)	0.0895	<0.01 (*)	0.2799	0.4599	0.3555

(*) represents statistically significant features within the 99% confidence interval.

**Table A2:** ANOVA statistical results of features in the θ band.

Channels	Features							
	MEA	ENE	VAR	RMS	PSD	CF	FV	MSF
FP1	0.9627	0.5251	0.5246	<0.01 (*)	0.5596	0.3307	<0.01 (*)	0.2448
FP2	0.9750	<0.01 (*)	<0.01 (*)	<0.01 (*)	<0.01 (*)	<0.01 (*)	<0.01 (*)	<0.01 (*)
F3	0.9888	<0.01 (*)	<0.01 (*)	0.7138	<0.01 (*)	0.0652	0.2383	<0.01 (*)
F4	0.9350	0.0672	0.0672	<0.01 (*)	0.3123	<0.01 (*)	0.0649	<0.01 (*)
C3	0.9748	<0.01 (*)	<0.01 (*)	<0.01 (*)	<0.01 (*)	<0.01 (*)	0.5767	<0.01 (*)
C4	0.9961	<0.01 (*)	<0.01 (*)	<0.01 (*)	<0.01 (*)	<0.01 (*)	<0.01 (*)	<0.01 (*)
P3	0.9610	<0.01 (*)	<0.01 (*)	0.6929	<0.01 (*)	0.4533	0.9495	0.3834
P4	0.9595	<0.01 (*)	<0.01 (*)	<0.01 (*)	<0.01 (*)	0.8968	0.1558	0.8600
O1	0.9859	<0.01 (*)	<0.01 (*)	<0.01 (*)	<0.01 (*)	<0.01 (*)	<0.01 (*)	<0.01 (*)
O2	0.9889	<0.01 (*)	<0.01 (*)	<0.01 (*)	<0.01 (*)	<0.01 (*)	0.2881	<0.01 (*)
F7	0.9550	<0.01 (*)	<0.01 (*)	<0.01 (*)	0.0686	0.1455	<0.01 (*)	0.0812
F8	0.9887	<0.01 (*)	<0.01 (*)	<0.01 (*)	<0.01 (*)	0.2455	0.4559	0.2359
T3	0.9778	<0.01 (*)	<0.01 (*)	<0.01 (*)	<0.01 (*)	<0.01 (*)	<0.01 (*)	<0.01 (*)
T4	0.9726	<0.01 (*)	<0.01 (*)	<0.01 (*)	<0.01 (*)	0.3887	<0.01 (*)	0.6247
T5	0.9933	<0.01 (*)	<0.01 (*)	0.8864	<0.01 (*)	0.8727	<0.01 (*)	0.6848
T6	0.9912	0.6568	0.6566	<0.01 (*)	0.9070	<0.01 (*)	0.4994	<0.01 (*)

(*) represents statistically significant features within the 99% confidence interval.

**Table A3:** ANOVA statistical results of features in the α band.

Channels	Features							
	MEA	ENE	VAR	RMS	PSD	CF	FV	MSF
FP1	0.9692	0.1182	0.1183	<0.01 (*)	0.1534	<0.01 (*)	0.8561	<0.01 (*)
FP2	0.9751	<0.01 (*)	<0.01 (*)	<0.01 (*)	<0.01 (*)	<0.01 (*)	0.0519	<0.01 (*)
F3	0.9458	0.4699	0.4700	0.0518	0.5761	<0.01 (*)	0.6728	<0.01 (*)
F4	0.9145	<0.01 (*)	<0.01 (*)	<0.01 (*)	0.3642	0.2280	<0.01 (*)	0.2551
C3	0.9469	<0.01 (*)	<0.01 (*)	<0.01 (*)	<0.01 (*)	0.8638	<0.01 (*)	0.9240
C4	0.8831	<0.01 (*)	<0.01 (*)	<0.01 (*)	<0.01 (*)	<0.01 (*)	0.5363	<0.01 (*)
P3	0.9899	<0.01 (*)	<0.01 (*)	<0.01 (*)	<0.01 (*)	<0.01 (*)	0.2360	<0.01 (*)
P4	0.9403	0.2499	0.2504	<0.01 (*)	0.5821	0.2738	0.5165	0.2631
O1	0.9779	<0.01 (*)	<0.01 (*)	<0.01 (*)	<0.01 (*)	<0.01 (*)	<0.01 (*)	<0.01 (*)
O2	0.9459	<0.01 (*)	<0.01 (*)	<0.01 (*)	<0.01 (*)	<0.01 (*)	<0.01 (*)	<0.01 (*)
F7	0.9364	0.1537	0.1537	0.0610	0.2236	<0.01 (*)	0.0654	<0.01 (*)
F8	0.9483	0.3195	0.3196	<0.01 (*)	0.3951	<0.01 (*)	0.6090	<0.01 (*)
T3	0.9429	<0.01 (*)	<0.01 (*)	<0.01 (*)	<0.01 (*)	<0.01 (*)	0.4380	<0.01 (*)
T4	0.9591	<0.01 (*)	<0.01 (*)	<0.01 (*)	<0.01 (*)	<0.01 (*)	0.3023	<0.01 (*)
T5	0.9602	0.1594	0.1595	0.3931	0.2193	0.5166	0.7923	0.4852
T6	0.9826	<0.01 (*)	<0.01 (*)	<0.01 (*)	0.1421	0.9477	0.8905	0.8945

(*) represents statistically significant features within the 99% confidence interval.

**Table A4:** ANOVA statistical results of features in the β band.

Channels	Features							
	MEA	ENE	VAR	RMS	PSD	CF	FV	MSF
FP1	0.9796	0.1488	0.1488	0.1846	<0.01 (*)	<0.01 (*)	<0.01 (*)	<0.01 (*)
FP2	0.9829	<0.01 (*)	<0.01 (*)	<0.01 (*)	<0.01 (*)	<0.01 (*)	0.1145	<0.01 (*)
F3	0.9786	0.0785	0.0785	0.4634	0.1338	<0.01 (*)	0.6512	<0.01 (*)
F4	0.9912	0.5586	0.5585	<0.01 (*)	0.3192	<0.01 (*)	0.9168	<0.01 (*)
C3	0.9653	<0.01 (*)	<0.01 (*)	<0.01 (*)	<0.01 (*)	<0.01 (*)	<0.01 (*)	<0.01 (*)
C4	0.9722	<0.01 (*)	<0.01 (*)	<0.01 (*)	<0.01 (*)	<0.01 (*)	<0.01 (*)	<0.01 (*)
P3	0.9965	<0.01 (*)	<0.01 (*)	<0.01 (*)	<0.01 (*)	<0.01 (*)	<0.01 (*)	<0.01 (*)
P4	0.9449	<0.01 (*)	<0.01 (*)	<0.01 (*)	0.3553	<0.01 (*)	0.6807	<0.01 (*)
O1	0.9605	<0.01 (*)	<0.01 (*)	<0.01 (*)	<0.01 (*)	<0.01 (*)	<0.01 (*)	<0.01 (*)
O2	0.9521	<0.01 (*)	<0.01 (*)	<0.01 (*)	<0.01 (*)	<0.01 (*)	<0.01 (*)	<0.01 (*)
F7	0.9527	0.5608	0.5608	0.8829	0.7697	<0.01 (*)	0.6108	<0.01 (*)
F8	0.9773	<0.01 (*)	0.0259	<0.01 (*)	0.1056	<0.01 (*)	0.7110	<0.01 (*)
T3	0.9781	<0.01 (*)	<0.01 (*)	<0.01 (*)	<0.01 (*)	<0.01 (*)	<0.01 (*)	<0.01 (*)
T4	0.9059	<0.01 (*)	<0.01 (*)	<0.01 (*)	<0.01 (*)	<0.01 (*)	<0.01 (*)	<0.01 (*)
T5	0.9921	<0.01 (*)	<0.01 (*)	0.1721	<0.01 (*)	<0.01 (*)	<0.01 (*)	<0.01 (*)
T6	0.9719	0.4500	0.4501	<0.01 (*)	0.2209	<0.01 (*)	0.3089	<0.01 (*)

(*) represents statistically significant features within the 99% confidence interval.
